# The impact of comprehensive licensure review on nursing students’ clinical competence, self-efficacy, and work readiness

**DOI:** 10.1016/j.heliyon.2024.e28506

**Published:** 2024-03-26

**Authors:** Adnan Innab, Monir M. Almotairy, Naji Alqahtani, Ahmed Nahari, Reem Alghamdi, Hamza Moafa, Dalal Alshael

**Affiliations:** a Nursing Administration and Education Department, College of Nursing, King Saud University, Riyadh, 12372, Saudi Arabia; b Medical Surgical Nursing Department College of Nursing, King Saud University, Riyadh, 12372, Saudi Arabia; c Maternity and Child Health Nursing Department, College of Nursing, King Saud University, Riyadh, 12372, Saudi Arabia; d Community and Psychiatric Mental Health Nursing College of Nursing, King Saud University, Riyadh, 12372, Saudi Arabia

**Keywords:** Computerized adaptive testing, Nursing licensure, Clinical competence, Self-efficacy, Work readiness

## Abstract

This study aims to assess the effectiveness of comprehensive licensure reviews and adaptive quizzing assignments on nursing students' clinical competence, self-efficacy, and work readiness—an under-researched topic. Additionally, it seeks to explore the mediating effect of self-efficacy in the relationship between students’ clinical competence and work readiness. A quasi-experimental (pre- and post-test), single-group design was employed. The study was conducted in a public university in Saudi Arabia and included a total of 293 senior nursing students in their last year of the bachelor program. An intervention was developed based on the blueprints of the Saudi Nursing Licensing Exam and NCLEX-RN and consisted of a weekly 3-h synchronous comprehensive licensure review bundled with 23 adaptive quizzing assignments over 15 weeks. Data were collected prior to and after the intervention using three scales: clinical competence, self-efficacy, and work readiness. The mean scores of clinical competence, self-efficacy, and two subscales of work readiness (work competence and social intelligence) increased significantly post-intervention.

Self-efficacy (β = 0.353, *p* < 0.001) and clinical competence (β = 0.251, *p* < 0.001) influenced work readiness (*F* [5, 226] = 21.03, *p* < 0.001) and accounted for 31.8% of the explained variability in work readiness. In the mediation analysis, clinical competence had a significant and indirect effect on work readiness through self-efficacy (B = 0.464, *p* < 0.001, 95% CI 0.250 to 0.699). The proportion of mediation indicated that 37.2% of the total effect of clinical competence on work readiness was due to the indirect effect of self-efficacy. Comprehensive licensure review and adaptive quizzing assignments improve students’ perceptions of clinical competence and self-efficacy. Such interventions could ease the transition of senior nursing students to clinical practice.

## 1 Introduction

The need for highly competent and qualified graduate nurses to mitigate the shortage in the nursing workforce is greater than ever before [1]. The nursing shortage poses a major obstacle to healthcare systems across the globe and has necessitated national calls to increase the number of nursing student admissions [1]. Therefore, it is expected that the number of newly graduated nurses will increase dramatically in the next few years to meet the projected demand for additional nurses in Saudi Arabia and across the world. These new graduate registered nurses (NGRNs), despite being novices, are expected to take on the role of registered nurses and demonstrate competence in handling patient workloads, executing nursing skills, and effectively prioritizing nursing activities. However, a global debate on their readiness for clinical practice persists [2,3]. For example, across the globe, there is a substantial turnover of NGRNs during their first year of practice, as many novice nurses fall short of these requirements [4].

In recent years, the clinical competency of NGRNs has become a concern for nurse academics, clinical leaders, and policymakers [5,6]. Research suggests that there is a decline in the clinical competency of NGRNs, which may potentially harm patients and lead to several negative health outcomes [7,8]. For example, only 14 percent of NGRNs in the United States demonstrated acceptable entry-level competencies and practice readiness in 2020 compared to 23 percent in 2015 [7,8]. Therefore, special consideration should be given to students as they approach graduation to ensure that they acquire entry-level competencies, especially during the last year of the nursing school program for a successful transition into a professional position. In this regard, Nash et al. stated that the final year acts as a pre-transition process and enables nursing students to “build their clinical confidence and consolidate their clinical skills, while also developing positive professional qualities and work attitudes” [9]. Therefore, the success or failure of the transition process depends on the school support offered to pre-transition students as they prepare for a professional nursing job.

The state of preparedness for a job as a professional nurse is termed “work readiness,” and refers to the perceived competence and attributes of graduates when they enter the workforce [10]. Other researchers have conceptualized it as “a state of mind” that reflects graduates' ability to manage both the responsibilities of the role and their workplace behavior while performing their jobs [11]. Nursing students' readiness for the workforce is influenced by educational factors, such as professional competence and clinical practice, and personal factors, such as students' background and feelings [12]. Therefore, to improve students' preparedness for practice, it has been argued that nursing educators should strive to enhance students' clinical competence, defined as the ability to execute tasks with desirable outcomes under diverse real-world circumnstances [13]. Evidence has shown that nursing students with higher levels of perceived clinical competence display higher levels of self-efficacy [14,15]. Self-efficacy reflects an individual's belief in their capacity to achieve a particular goal [16]. It has been shown that self-efficacy is a good predictor of nursing student performance in clinical practice, and that new graduate nursing students need a high level of self-efficacy to feel capable of meeting entry-level clinical standards and embracing challenging responsibilities [4]. Consequently, to enhance and foster newly graduated nurses' work readiness, nursing educators have been encouraged to develop and incorporate effective teaching pedagogies into nursing curricula that develop clinical competency and self-efficacy [[17], [18], [19]].

A wide range of innovative teaching approaches, including refresher courses, simulation-based learning, clinical teaching models, micro learning, interactive education, preceptorship programs, and intensive clinical skill courses, has been used to improve nursing students' clinical competence, self-efficacy, and work readiness [15,[20], [21], [22], [23], [24], [25]]. In Saudi Arabia, researchers found that simulation-based training improves students' self-efficacy and clinical competence [26]. Some teaching approaches use a combination of a comprehensive licensure review and adaptive quizzing assignments, serving as a formative assessment tool [27], to improve the performance of undergraduate nursing students in the end-of-program exit exam [28]. However, little is known about the effectiveness of comprehensive licensure reviews and adaptive quizzing assignments on students’ perceptions of their clinical competence, self-efficacy, and work readiness in Saudi Arabia.

Accordingly, this study aimed to (1) evaluate the influence of comprehensive licensure review and adaptive quizzing assignments on nursing students' clinical competence, self-efficacy, and work readiness; (2) identify the association between clinical competence, self-efficacy, and work readiness; and (3) investigate the mediating role of self-efficacy in the relationship between students’ clinical competence and work readiness.

## 2 Materials and methods

### 2.1 Theoretical framework

This study was grounded in Bandura's theory of self-efficacy, which contends that individuals with a high perceived self-efficacy are more likely to master a given task compared to those with low perceived self-efficacy [16]. Bandura identified four distinct sources of an individual's sense of self-efficacy: performance achievement, vicarious experiences, verbal persuasion, and physiological arousal. The perceived clinical competence of students is associated with performance achievement, while encouragement from faculty members during the intervention aligns with verbal persuasion. By enhancing these sources, an improvement in students' work readiness is anticipated, reflecting the desired outcome within the framework of the theory.

### 2.2 Study design

This study employed a quasi-experimental (pre- and post-test) single-group design. The research team followed the TREND statement checklist for nonrandomized controlled trials. This study was approved by the Ethics Committee of [King Saud University], with ethics approval reference [KSU-HE-22-001]). Informed consent was obtained from all the participants prior to data collection. Participants were informed that their participation was voluntary and their information would be kept strictly confidential.

### 2.3 Sample and settings

Nursing students were recruited from a public university in Saudi Arabia. Data were collected at two time points using convenience sampling. The inclusion criteria were senior students who were (1) in the last semester of the bachelor program and (2) enrolled in the nursing review course during the study period. The sample size required to run the statistical analysis was determined using G-power software. At a significance level of 0.05, power of 0.80, and effect size of 0.15, a minimum sample of 123 participants was required to perform the bivariate and multivariate analysis. The total sample size of this study was 293.

### 2.4 The study intervention

Students received a comprehensive licensure review bundled with adaptive quizzing assignments over a 15-week semester. The intervention included a comprehensive review of topics that address the blueprints of the Saudi Nursing Licensing Examination (SNLE) and National Council Licensure Examination for Registered Nurses (NCLEX-RN). The content of these topics was designed by the study authors to enhance students' knowledge of essential nursing content, enabling them to provide safe and high-quality nursing care (Table 1). The study investigators laid out the categories of topics in the blueprint of SNLE and NCLEX-RN, matched the topics into these categories, and structured each topic to address pathophysiology, common and complex health conditions, and proper nursing practice to promote health outcomes. The review content was delivered to enhance students’ cognitive skills to prioritize health needs, deliver the proper nursing interventions, and evaluate the effectiveness of these interventions. Furthermore, the content incorporated relevant ethical principles and professional values that nurses are expected to uphold in their daily activities. The intervention was delivered on a weekly basis during scheduled morning and early afternoon sessions.

**Table 1:** Timeline for the comprehensive licensure review, adaptive quizzing assignments, and data collection.

Course Content (3 h weekly)	Delivery of the Intervention Bundle	Adaptive Quizzing Assignments	Data Collection (Week 1 & 15)
Medication calculations.	• Test taking strategies. • Case Studies. • Scenarios. • Videos, animations, infographic, and audio materials on anatomy, physiology, pathophysiology, clinical manifestation, diagnostics procedures, multidisciplinary management approaches, pharmacological and non-pharmacological management. • Practicum Q&An on the weekly content.	Weekly activities to achieve a mastery level of 8 in 23 topics relevant to weekly content. These topics were: 1. Acid-Base Balance. 2. Fluid & Electrolyte Balance. 3. Medication Calculations. 4. Gastrointestinal Disorders. 5. Endocrine and Metabolic Disorders. 6. Respiratory Disorders. 7. Cardiovascular Disorders. 8. Genitourinary Disorders. 9. Neurosensory Disorders. 10. Immune and Hematologic Disorders. 11. Oncologic Disorders. 12. Musculoskeletal Disorders. 13. Antepartum Period. 14. Intrapartum Period. 15. Postpartum Period. 16. The Neonate. 17. Infant. 18. Toddler. 19. Preschooler. 20. School-age Child. 21. Adolescent. 22. Psychiatric and Mental Health Nursing. 23. Prioritization and Delegation.	Demographic characteristics online survey Work readiness, clinical competence, and self-efficacy survey.
Fluid & Electrolytes/Acid Base Balance.			
Cardiovascular System-1.			
Cardiovascular System-2.			
Respiratory System.			
Neurological System.			
Renal System.			
Musculoskeletal System.			
Endocrine System.			
Immune, Hematology, and Oncology.			
Gastrointestinal System**.**			
Special Topics for Women Health**.**			
Special Topics for Pediatric and Adolescent Health.			
Community and psychiatric.			
Unit management and leadership.			Work readiness, clinical competence, and self-efficacy survey.

Furthermore, the intervention comprised 23 adaptive quizzing assignments, distributed over 15 weeks, utilizing a validated adaptive quizzing system. The adaptive quizzing system delivered NCLEX-RN-type questions and evaluated students' performance using mastery levels ranging from level 1 (lowest level) to 8 (highest level). The transition between levels depended on students’ sustained performance [29].

To ensure intervention fidelity, the study investigators used the NCLEX-RN comprehensive review textbooks to develop the intervention content material, which were deemed as appropriate sources for the Saudi context based on the SNLE blueprint. The adaptive quizzing assignments were performed through an authenticated and validated source that has been used by prelicensure students to prepare for NCLEX-RN examinations [29].

## 3 Study measures

### 3.1 Demographic measures

Demographic measures included age, gender, and grade point average (GPA).

#### 3.1.1 Clinical competence

Clinical competence was measured using the Clinical Competence Questionnaire (CCQ), which comprises 47 items and 2 subscales: professional nursing behavior and skill competence. Skill competence consists of three domains: general performance, core nursing skills, and advanced nursing skills [30]. The items are rated on a 5-point Likert scale ranging from 1 (*do not have a clue*) to 5 (*known in theory, competent in practice without supervision*). The overall scores range from 47 to 235, with higher scores indicating a greater level of competence [31]. The entire scale was tested and showed excellent reliability (Cronbach's α = 0.98).

#### 3.1.2 Self-efficacy

Students' self-efficacy was measured using the Generalized Self-Efficacy (GSE) scale, which has been widely used in adult populations [32]. It is a unidimensional scale consisting of 10 items rated on a 4-point Likert scale ranging from 1 (*not at all*) to 4 (*exactly true*). The scores range from 10 to 40, with higher scores indicating greater self-efficacy. The reliability of the scale was tested (Cronbach's α ranged from 0.76 to 0.90).

#### 3.1.3 Work readiness

Students' work readiness was measured using the Work Readiness Scale-Graduate Nurse (WRS-GN). This scale consists of 46 items and 4 subscales: personal characteristics, organizational acumen, work competence, and social intelligence [10]. The items are rated on a 10-point Likert scale, ranging from 1 (*completely disagree*) to 10 (*completely agree*). The reliability was tested and revealed that the instrument had good internal consistency (Cronbach's α = 0.92). Cronbach's α for the subscales ranged from 0.84 to 0.88, indicating acceptable reliability across the subscales [10].

## 4 Data collection

Data were collected during the pre- and post-intervention phases. In the former phase, an online survey was distributed to students to collect data on their demographic characteristics, work readiness, clinical competence, and self-efficacy. During the first week, students received two reminders encouraging them to participate in the surveys at their convenience. After completing the intervention, students participated in the post-intervention survey to collect data on their work readiness, clinical competence, and self-efficacy. The post-intervention online survey was completed at the students’ convenience during the final week of the intervention. Their email addresses were used to link the pre- and post-intervention data.

### 4.1 Data analysis

Data were analyzed using IBM SPSS Statistics for Windows version 28. The central tendency (e.g., mean and median) and dispersion (e.g., standard deviation and variance) measures were used to present descriptive results. Paired-sample t-tests were used to determine the differences in students’ clinical competence, self-efficacy, and work readiness before and after the intervention. A multiple linear regression analysis was used to determine whether clinical competence and self-efficacy predicted work readiness, while controlling for the effect of demographic characteristics. Process macro model 4 and bootstrapping were used to determine the mediating effect of self-efficacy in the relationship between clinical competence and work readiness [33].

## 5 Results

### 5.1 Demographic characteristics of participants

Participants ranged in age from 21 to 27 years, with an average of 22.4 years (SD ± 0.94). The majority (55.5%) of participants were female. The mean students’ GPA was 4.1 (SD ± 0.40).

### 5.2 Mean differences pre- and post-intervention

Paired-sample t-tests were used to determine the difference in the mean scores of clinical competence, self-efficacy, and work readiness before and after the intervention (Table 2). The mean score of clinical competence was 164.2 (SD = 38.1) pre-intervention, which increased significantly to 172.5 (SD = 38.0) post-intervention (t = 3.22, *p* < 0.001), indicating that students perceived themselves to be competent in performing nursing skills, but under supervision (Table 2). Specifically, significant improvement was noted in students’ general performance (*p* = 0.04), core nursing skills (*p* = 0.001), and advanced nursing skills (*p* < 0.001) post-intervention. Additionally, nursing students showed a moderate sense of self-efficacy pre-intervention (M = 29.8, SD = 5.9) but significantly higher self-efficacy post-intervention (M = 30.9, SD = 6.0, t = 2.89, *p* = 0.004). Regarding work readiness, a significant increase was noted in the mean scores of the work competence (*p* = 0.023) and social intelligence (*p* = 0.019) subscales only. The *t*-test results showed no statistically significant differences between the pre-intervention (M = 343.9, SD = 86.4) and post-intervention (M = 340.2, SD = 88.4) mean scores of the entire work readiness scale.

**Table 2:** Comparison of students’ self-efficacy, clinical competence, and work readiness (N = 292).

Variables	Pre-test	Post-test	*t*	*p*	*95% CI*	
	M (*SD*)	M (*SD*)			Lower	Upper
Clinical Competence	164.2 (38.1)	172.5 (38.0)	*3.22*	<0.001	*3.27*	13.50
Nursing Professional Behavior	61.9 (14.5)	62.9 (13.9)	0.919	0.359	−1.02	2.82
SC: General performance	47.1 (11.9)	48.8 (11.6)	2.02	0.044	0.047	3.28
SC: Core nursing skills	38.7 (10.6)	41.2 (10.4)	3.31	0.001	0.962	3.79
SC: Advanced nursing skills	16.3 (5.6)	19.7 (5.7)	8.66	<0.001	2.65	4.21
**Self-Efficacy**	**29.8 (5.9)**	**30.9 (6.0)**	**2.89**	0***.004***	0**.358**	**1.89**
**Work Readiness**	**343.9 (86.4)**	**340.2 (88.4)**	0**.562**	0**.575**	***−9.31***	***16.75***
Personal Work Characteristics	57 (15.4)	52.8 (17.3)	−3.32	0.001	*−6.70*	*−1.71*
Organizational Acumen	128.4 (38.5)	127.6 (37.4)	−0.275	0.784	*−6.48*	*4.88*
Work Competence	106.1 (34.1)	112 (34.7)	2.28	0.023	0*.80*	*10.9*
Social Intelligence	48.5 (16.8)	51.4 (17.0)	2.36	0.019	0*.47*	*5.2*

Notes: SC, Skill Competence; M: Mean; SD: Standard Deviation; CI: Confidence Interval.

## 6 The influence of self-efficacy and clinical competence on work readiness

Multiple linear regression was used to determine the influence of self-efficacy and clinical competence on work readiness pre- and post-intervention, while controlling for demographic characteristics (Table 3). The first model (pre-intervention) was significant (*F* [5, 226] = 26.76, *p* < 0.001, *R*^2^ = 0.372). Self-efficacy (β = 0.308, *p* < 0.001) and clinical competence (β = 0.352, *p* = <0.001) statistically significantly predicted work readiness. Post-intervention, both self-efficacy (β = 0.353, *p* < 0.001) and clinical competence (β = 0.251, *p* < 0.001) remained significant predictors of work readiness (*F* [5, 226] = 21.03, *p* < 0.001) and accounted for 31.8% of the explained variability in work readiness.

**Table 3:** The influence of nursing students’ self-efficacy and clinical competence on work readiness.

Variables	Work Readiness											
	Pre-test						Post-test					
	B^a^	β^b^	*t*	*p*	95% CI		B^a^	β^b^	*t*	*p*	95% CI	
					Lower	Upper					Lower	Upper
Self-Efficacy	1.16	0.308	4.36	**<**0**.001**	0.632	1.677	1.13	0.353	4.94	**<**0**.001**	0.683	1.588
Clinical Competence	0.825	0.352	5.75	**<**0**.001**	0.497	1.15	0.521	0.251	3.55	**<**0**.001**	0.232	0.811
Age	1.529	0.065	1.20	0.229	−0.969	4.02	−0.140	−0.006	−0.112	0.911	−2.60	2.32
Gender	−0.481	−0.011	−0.187	0.851	−5.54	4.57	−1.53	−0.036	−0.603	0.547	−6.53	3.48
GPA	−4.750	−0.078	−1.35	0.177	−11.66	2.16	5.85	0.101	1.68	0.094	−1.00	12.70
Model Summary	F(5, 226) = 26.76, R^2^ = 0.372, *p* < 0.001						F(5, 226) = 21.03, R^2^ = 0.318, *p* <. 001					

Multiple Linear Regression was used. Dependent Variable: Work Readiness.

B^a^ Unstandardized beta coefficient.

B^b^ Beta standardized coefficient.

## 7 The mediating role of self-efficacy on the relationship between clinical competence and work readiness

**Fig. 1:**
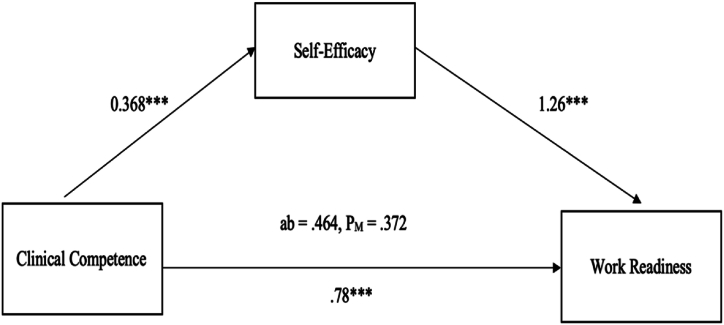
The mediating role of self-efficacy on the relationship between clinical competence and work readiness.

Table 4 depicts the mediating role of self-efficacy in the relationship between clinical competence and work readiness. The results indicate that clinical competence had a direct effect on work readiness (B = 0.78, *p* < 0.001, 95% CI: 0.514–1.049) and a significant effect on self-efficacy (B = 0.368, *p* < 0.001, 95% CI 0.311 to 0.425), which, in turn, had a significant effect on work readiness (B = 1.26, p < 0.001, 95% CI 0.827 to 1.692). Thus, clinical competence had a significant and indirect effect on work readiness through self-efficacy (B = 0.464, *p* < 0.001, 95% CI 0.250 to 0.699). The proportion of mediation (P_M_ = 0.372) indicated that 37.2% of the total effect of clinical competence on work readiness was due to the indirect effect of self-efficacy, which was considered partial mediation (Fig. 1).

**Table 4:** Results of mediating effect of self-efficacy by bootstrapping (N = 139).

Effect	Variables	B	SE	t	*p*	95% CI		P_M_
						**LLCI**	**ULCI**	
**Direct Effect**	Clinical competence → Work Readiness (c′)	0.78	0.136	5.75	0***.000***	0.514	1.049	0.372
**Indirect Effect**	Clinical Competence → Self-efficacy (a)	0.368	0.029	12.66	0**.000**	0.311	0.425	
**Indirect Effect**	Self-efficacy → Work Readiness (b)	1.26	0.219	5.73	0**.000**	0.827	1.692	
**Indirect Effect**	Clinical competence → Self-efficacy → Work Readiness (ab)	0.464	0.114			0.250	0.699	
**Total Effect**	(c′+ab)	1.246	0.115	10.85	0**.000**	1.020	1.472	

Note: 5000 bootstrapping re-extracted.

B=B coefficient, SE = Standard of Error, CI = Confidence Interval, LLCI = lower limit of B in 95% CI, ULCI = upper limit of B in 95% CI, P_M_ = proportion mediated, the ratio of the indirect effect to the total effect.

## 8 Discussion

This study evaluated Saudi senior nursing students' perceptions of their clinical competence, self-efficacy, and work readiness before and after receiving the comprehensive licensure review and adaptive quizzing assignments intervention. The findings revealed that the intervention significantly improved students’ perceived clinical competence and self-efficacy; however, it did not significantly improve their overall perceptions of work readiness.

First, with respect to clinical competence, the CCQ subscale analyses revealed that the intervention significantly improved the students' skill competence subscale domain scores, including general performance, core nursing skills, and advanced nursing skills. Although the intervention did not significantly enhance students’ professional nursing behavior scores, they were acceptable for their level and comparable to those of a previous study among Saudi senior nursing students [32]. The professional nursing behavior subscale had the highest mean, followed by the general performance, core nursing skills, and advanced nursing skills subscales. Similar findings have been reported among Saudi nursing interns and senior nursing students [15,20]. However, contrary to the findings of this study, another study among Saudi senior nursing students revealed that core nursing skills had the highest mean, followed by general performance skills, advanced nursing skills, and professional nursing behaviors [14]. These variations in findings regarding nursing clinical competencies among studies in Saudi Arabia may be attributed to differences in study designs, resources, and the content of nursing programs delivered across the country.

Second, the findings revealed that the intervention significantly improved students' self-efficacy. The students exhibited a high level of self-efficacy, consistent with previous studies conducted in Saudi Arabia among senior nursing students [14,15]. This could be linked to enhanced levels of clinical competence gained from the intervention. In this study, students perceived themselves as clinically competent, which is consistent with results reported in two previous studies [30,34]. Additionally, clinical competence was positively associated with self-efficacy, which also aligned with previous findings among senior Saudi nursing students [14,15]. Another possible explanation could be the educational experience and quality of the intervention, which utilized course-specific adaptive and interactive learning, taking into consideration students’ performance differences.

Third, the intervention did not significantly improve students' overall perceptions of work readiness; however, it did significantly enhance their scores on work competence and social intelligence subscales. Nevertheless, students scored relatively high on overall work readiness and its subscales pre- and post-intervention. Prior research has consistently reported lower levels of work readiness among newly graduated nurses in various countries, including Saudi Arabia [17], China [35], and Australia [23]. However, the level of work readiness was higher among the same group in the United States [19]. The high overall work readiness scores in the current study could be attributed to students’ excitement and eagerness to begin their careers.

Furthermore, the improved scores on the work competence and social intelligence subscales can be attributed to the intervention, which included comprehensive case studies representing real-life situations faced by practicing nurses, possibly increasing senior nursing students' sense of confidence related to perceived work competence and social skills. A previous study among newly graduated Saudi nursing interns reported that those who began training had higher perceptions of work competence and social intelligence than those who did not [17]. This suggests that the intervention engaged students in activities that were comparable to the real work environment. However, it also diminished students' perceptions of their personal work characteristics subscale scores, perhaps because the intervention is a new and innovative method of teaching compared to traditional teaching approaches. Dudley and colleagues [21] concluded that lower perceived work readiness, including personal work characteristics, work competence, social intelligence, and organizational acumen subscales, was associated with innovative teaching methods. Therefore, further research is needed to investigate the elements of innovative education that enhance and diminish nurses' overall work readiness. Prior studies indicated that newly graduated nurses’ perception of personal work characteristics reduces after they begin training [17,21]. A possible explanation for the reduced scores in this study could be the job reality shock of the intervention, which provided an experience similar to actual nursing practice.

First, perceived clinical competence was positively associated with and influenced perceived work readiness pre- and post-intervention. Although prior literature has not explored the relationship between clinical competence and work readiness, perception of clinical competence is essential to prepare individuals for work, as clinically competent graduate nurses expressed satisfaction with their work [36]. Other researchers concluded that having clinical competence is a predictor of higher levels of compassion satisfaction [37]. In line with the results of this study, Zakeri et al. [38] found a significant positive relationship between compassion satisfaction and clinical competence, indicating that nurses' clinical competence could increase with higher levels of satisfaction. Therefore, understanding the stressors that affect student nurses can be beneficial in improving nurses' competence levels. This would help nursing students perceive themselves as confident in their knowledge and believe that they have the requisite level of professional competence. By addressing negative factors, student nurses' performance and career optimization can be improved, ultimately leading to an increase in their clinical competence. In short, it is crucial to acknowledge students’ feedback and the emotional impact on the development of their self-efficacy and work readiness.

Second, perceived self-efficacy was positively associated with and influenced perceived work readiness pre- and post-intervention. Furthermore, perceived self-efficacy partially mediated the relationship between perceived clinical competence and work readiness. Therefore, students felt competent with their skills and had confidence in their ability to prepare for their nursing careers. This finding is in line with Bandura's self-efficacy theory, where mastery experiences, represented by clinical competence as a source of self-efficacy, influence the desired behavior, namely work readiness [16]. To the best of the authors' knowledge, this is the first study to examine the mediating effect of self-efficacy in the relationship between clinical competence and work readiness.

## 9 Implications and recommendations

The findings of this study have important implications for nursing academic administrators and educators in not only improving students' clinical competence and self-efficacy but also facilitating their work readiness. Academic programs may be beneficial by adopting a comprehensive review course and adaptive quizzing assignments to enhance students' perceptions of clinical competence and self-efficacy, ultimately improving their work readiness. The integrated activities included in the intervention represented real-life situations faced by practicing nurses. Therefore, the inclusion of such activities throughout the nursing curricula and courses could foster students' knowledge, critical thinking, clinical judgment, and reasoning skills related to clinical practice. Based on the results of this study, improving nursing students’ clinical competence and self-efficacy, through course trainings and practice, might be taken into consideration in nursing programs to strengthen work readiness across Saudi Arabia. Therefore, future studies should explore not only the mediating effects of self-efficacy on aspects related to work readiness but also the effect of job reality shock on work readiness among nursing graduates.

## 10 Strengths and limitations

This study demonstrates the strength and effectiveness of a structured intervention in enhancing clinical competence, self-efficacy, and two subscales of work readiness. The findings provide significant contributions to comprehending the influence of structured educational interventions on the readiness of nursing students. Furthermore, they underscore the importance of tailored educational strategies in nursing education, emphasizing their role in preparing students for clinical practice.

Nonetheless, this study has some limitations. First, students’ clinical competence, self-efficacy, and work readiness were self-reported. Therefore, participants themselves may have given unrealistic scores, which could affect the accuracy and objectivity of the evaluation method. Another limitation is the convenience sample, which may reduce the generalizability of the findings to graduates from other universities. A large-scale study should be conducted in the future that will include participants from different universities to reach generalizable conclusions. Additionally, the quasi-experimental design resulted in the absence of a control group, preventing the assessment of the net effect of the intervention compared to a standard learning process, which could potentially be addressed in future research.

## 11 Conclusions

The implementation of a comprehensive review course and adaptive quizzing assignments improved students' perceptions of clinical competence and self-efficacy. Students' clinical competence and self-efficacy positively influenced their perceptions of work readiness. Such interventions could ease the transition of senior nursing students to clinical practice. Therefore, programs should be developed and implemented to not only boost students’ perceptions of clinical competence and self-efficacy but also enhance their work readiness.

## Data availability statement

The data that support the findings of this study are available from the corresponding author, [AI], upon reasonable request.

## Funding Sources

This study was funded by the Researchers Supporting Project (number:RSPD2024R837) of 10.13039/501100002383King Saud University, Riyadh, Saudi Arabia.

## CRediT authorship contribution statement

**Adnan Innab:** Writing – review & editing, Writing – original draft, Supervision, Methodology, Funding acquisition, Formal analysis, Data curation, Conceptualization. **Monir M. Almotairy:** Writing – review & editing, Writing – original draft, Software, Project administration, Methodology, Conceptualization. **Naji Alqahtani:** Writing – review & editing, Writing – original draft, Visualization, Resources, Investigation. **Ahmed Nahari:** Writing – review & editing, Writing – original draft, Visualization, Methodology, Conceptualization. **Reem Alghamdi:** Writing – review & editing, Writing – original draft, Validation, Conceptualization. **Hamza Moafa:** Writing – review & editing, Writing – original draft, Investigation, Conceptualization. **Dalal Alshael:** Writing – review & editing, Writing – original draft, Funding acquisition.

## Declaration of competing interest

The authors declare that they have no known competing financial interests or personal relationships that could have appeared to influence the work reported in this paper.
