# Supplementary material for: The impact of comprehensive licensure review on nursing students’ clinical competence, self-efficacy, and work readiness
Source: Heliyon. 2024 Mar 26;10(7):e28506. doi: 10.1016/j.heliyon.2024.e28506 (PMC11002570; doi:10.1016/j.heliyon.2024.e28506)
Supplement: Multimedia component 1 [file mmc1.pdf]

Work Readiness

Use the scale below to rate the extent to which you agree with the following statements. The ***higher*** the rating, the ***more*** you agree with the statement and the ***lower*** the rating, the ***less*** you agree with the statement.

1	2	3	4	5	6	7	8	9	10
Completely Disagree									Completely Agree

1	I get stressed when there are too many things going on (R)	1	2	3	4	5	6	7	8	9	10
2	Approaching senior people at work is a weakness for me (R)	1	2	3	4	5	6	7	8	9	10
3	I sometimes experience difficulty starting tasks (R)	1	2	3	4	5	6	7	8	9	10
4	I feel that I am unable to deal with things when I have competing demands (R)	1	2	3	4	5	6	7	8	9	10
5	I am sometimes embarrassed to ask questions when I am not sure about something (R)	1	2	3	4	5	6	7	8	9	10
6	I become overwhelmed by challenging circumstances (R)	1	2	3	4	5	6	7	8	9	10
7	Juggling too many things at once is one of my weaknesses (R)	1	2	3	4	5	6	7	8	9	10
8	I don’t like the idea of change (R)	1	2	3	4	5	6	7	8	9	10
9	You can learn a lot from your colleagues	1	2	3	4	5	6	7	8	9	10
10	There is a lot to learn from employees who have worked at an organisation for years	1	2	3	4	5	6	7	8	9	10
11	You can learn a lot from long serving employees, even if they do not have a university degree	1	2	3	4	5	6	7	8	9	10
12	As an employee it’s important to have a sound understanding of organisational processes and protocols	1	2	3	4	5	6	7	8	9	10
13	It is important to learn as much as you can about the organisation	1	2	3	4	5	6	7	8	9	10
14	It’s important to respect your colleagues	1	2	3	4	5	6	7	8	9	10
15	At work it is important to always take responsibility for your decisions and actions	1	2	3	4	5	6	7	8	9	10
16	It is important to respect authority figures	1	2	3	4	5	6	7	8	9	10
17	I look forward to the opportunity to learn and grow at work	1	2	3	4	5	6	7	8	9	10
18	I am eager to throw myself into my work	1	2	3	4	5	6	7	8	9	10
19	I am always working on improving myself	1	2	3	4	5	6	7	8	9	10
20	An organisation’s values and beliefs forms part of its culture	1	2	3	4	5	6	7	8	9	10

21	I see all feedback as an opportunity for learning	1	2	3	4	5	6	7	8	9	10
22	I thrive on completing tasks and achieving results	1	2	3	4	5	6	7	8	9	10
23	I can't wait to start work and throw myself into a project	1	2	3	4	5	6	7	8	9	10
24	I am confident about my learnt knowledge and could readily answer clinical questions about my field	1	2	3	4	5	6	7	8	9	10
25	I have a solid theoretical understanding of my field of work	1	2	3	4	5	6	7	8	9	10
26	People approach me for original ideas	1	2	3	4	5	6	7	8	9	10
27	Now that I have completed my studies I consider myself clinically competent to apply myself to the field.	1	2	3	4	5	6	7	8	9	10
28	I know my strengths and weaknesses	1	2	3	4	5	6	7	8	9	10
29	I remain calm under pressure	1	2	3	4	5	6	7	8	9	10
30	I feel confident that I will be able to apply my learnt knowledge to the workplace	1	2	3	4	5	6	7	8	9	10
31	I know how to cope with multiple demands	1	2	3	4	5	6	7	8	9	10
32	Analysing and solving complex problems is a strength for me	1	2	3	4	5	6	7	8	9	10
33	Being among the best in my field is very important to me	1	2	3	4	5	6	7	8	9	10
34	One of my strengths is that I have an eye for detail	1	2	3	4	5	6	7	8	9	10
35	I consider myself to have a mature view of life	1	2	3	4	5	6	7	8	9	10
36	Adapting to different social situations is one of my strengths	1	2	3	4	5	6	7	8	9	10
37	Developing relationships with people is one of my strengths	1	2	3	4	5	6	7	8	9	10
38	Others would say I have an open and friendly approach	1	2	3	4	5	6	7	8	9	10
39	I can express myself easily	1	2	3	4	5	6	7	8	9	10
40	I am good at making impromptu speeches	1	2	3	4	5	6	7	8	9	10
41	I adapt easily to new situations	1	2	3	4	5	6	7	8	9	10
42	I find I am good at reading other people's body language	1	2	3	4	5	6	7	8	9	10
43	I communicate effectively with different patients	1	2	3	4	5	6	7	8	9	10
44	I recognise when I need to ask for help	1	2	3	4	5	6	7	8	9	10
45	I am always prepared for the unexpected to occur	1	2	3	4	5	6	7	8	9	10
46	When a crisis situation that needs my attention arises I can easily change my focus	1	2	3	4	5	6	7	8	9	10
