# Supplementary material for: The impact of comprehensive licensure review on nursing students’ clinical competence, self-efficacy, and work readiness
Source: Heliyon. 2024 Mar 26;10(7):e28506. doi: 10.1016/j.heliyon.2024.e28506 (PMC11002570; doi:10.1016/j.heliyon.2024.e28506)
Supplement: Multimedia component 2 [file mmc2.pdf]

ORIGINAL RESEARCH

Developing and validating the Clinical Competence Questionnaire: A self-assessment instrument for upcoming baccalaureate nursing graduates

Shwu-Ru Liou, Ching-Yu Cheng

School of Nursing, Chang Gung University of Science and Technology at Chiayi Campus, Chiayi, Taiwan.

Correspondence: Ching-Yu Cheng. Address: School of Nursing, Chang Gung University of Science and Technology at Chiayi Campus, 2 Chiapu Rd. West Sec. Putz, Chiayi, Taiwan. Email: chingyuus@gmail.com

Received: May 29, 2013 **Accepted:** August 1, 2013 **Online Published:** September 24, 2013
DOI: 10.5430/jnep.v4n2p56 **URL:** http://dx.doi.org/10.5430/jnep.v4n2p56

Abstract

Background: Although researchers have evaluated nurse competence in past studies, few have focused on the competence levels of nursing students immediately prior to graduation. Additionally, many of the competence scales were not supported with strong evidence of reliability or validity. The purpose of this study was to develop and test the psychometric properties of the Clinical Competence Questionnaire (CCQ) that measures the perceived clinical competence of upcoming baccalaureate nursing graduates.

Methods: The Clinical Competence Questionnaire was developed based on Patricia Benner's "From Novice to Expert" model. This developed instrument was evaluated in a cross-sectional study. A total of 340 baccalaureate students in their final semester of a 2-year RN-to-BSN program in Taiwan completed and returned the questionnaire. Out of the 340 students, data from 293 students who did not have work experience were used to test reliability and validity of the scale. The instrument was tested for content, construct, and criterion-related validity.

Results: The Cronbach's alpha for the entire CCQ was .98. Content and known-groups validity were confirmed. Principal component analysis showed a high degree of explanation of competence and revealed four components of competence: nursing professional behaviors, core nursing skills, general performance, and advanced nursing skills.

Conclusion: The results from our study indicate the CCQ demonstrates good reliability and validity for measuring the perceived clinical competence of upcoming baccalaureate nursing graduates. The CCQ is also a useful tool and is easy to administer for the self-assessment of nursing clinical competence. Study limitations and further recommendations for nursing are discussed.

Key words

Clinical competence, Baccalaureate nursing pregraduates, Instrument psychometric tests

1 Introduction

The current shortage of nurses is regarded as a global issue and remains a significant challenge for healthcare administrators and nurse educators. When hiring new nursing graduates to solve this critical situation, healthcare professionals must confront other problematic issues. One primary issue is whether new nurses are competent and ready for clinical practice.

Researchers report that new nurses not only lack adequate competence for working in a clinical environment, but are also perceived by nurse managers to lack in competent clinical skills [1-3]. In a survey conducted with 57,000 nurse leaders, only 25% were fully satisfied with the performance of new nursing graduates at the time of their initial employment [4]. The challenge for nurse educators, therefore, is to promote nursing students' competence before graduation to ensure the students' competence levels prior to their first entry into practice [1]. However, few studies have focused on measuring nursing students' competence immediately prior to their graduation. In addition, many competence scales lack strong evidence that indicates reliability, validity, or both [5, 6].

1.1 Description of the problem

In discussions of nursing competence-related issues, nursing experts and scholars have debated numerous questions about the process of ensuring competence. Questions frequently asked include the following: (a) which skills are most critical for beginning practice? (b) what level of competence should be established for the beginning level of nurses? (c) how should competence be assessed? and (d) how should competence be defined [1, 7, 8]? Clinical administrators and academic faculties have evaluated the quantitative and qualitative attributes of nurse competence [3, 5, 8-10]. However, most of the studies evaluated the competence of all nurses without differentiating between the nurses' varying years of working experiences. Some instruments did not differentiate between various levels of education, such as diploma or degree competencies [6]. The studies identifying essential nurse competencies were also mostly descriptive, had small convenience samples, focused on technical skills, were not developed based on theoretical models, and lacked reports of strong reliability and validity for instruments used to evaluate competence. In addition, the instruments were all used to measure competence for nurses already practicing in clinical sites rather than student nurses [5, 6, 11].

Benner's [13] "From Novice to Expert" model details five levels of nursing experience: (a) novice, (b) advanced beginner, (c) competent, (d) proficient, and (e) expert. Novices are beginners with no experience and need clear rules to help them perform. Advanced beginners demonstrate acceptable performance based on previous experience. Competent nurses apply analytical thinking in patient care. A nurse with two to three years of work experience, at least in similar day-to-day situations, can reach the competent stage. Proficient nurses perceive and understand situations as whole parts to improve their decision-making. Expert nurses exhibit performance that is fluid, flexible, and highly proficient. Each level in the "From Novice to Expert" model builds on the previous level as nurses' experiences are refined and then expanded. In Benner's view, newly graduated nurses are mostly at the novice level of nursing experience.

1.2 Purpose

Because of increased demand for quality care and nurse retention, today's healthcare environment requires entry-level nurses to rapidly progress from the novice level and function independently at a competent level. Healthcare administrators and academic faculty must be encouraged to follow the steps of developing nurse competency, devoting a longer period of time to cultivate the clinical skills of beginning nurses. Therefore, the purpose of our study was to develop and test the psychometric properties of a self-assessment instrument that identifies and measures the clinical competencies, which are essential for upcoming baccalaureate nursing graduates. The instrument will indicate whether the students adequately demonstrate competencies needed by new nurses. We theorized that the competence level of upcoming baccalaureate nursing graduates must be at least in the clinical novice stage to prepare them for the clinical skills needed by new nurses in hospital settings.

2 Methods

2.1 Phase I: Instrument development

In the first step of developing our instrument, the Clinical Competence Questionnaire, we reviewed current literature to learn more about the concepts of competence. Many researchers have developed definitions for nurse competence.

Benner [13] defined competence as the integration of fundamental knowledge, clinical ability, performance, and attitude in the context of a nursing situation. The American Association of Colleges of Nursing [14] characterized a series of core competencies that baccalaureate nursing graduates must possess for entry into practice: critical thinking, communication, assessment, and technical skills. The Texas Higher Education Coordinating Board identified knowledge, judgment, skills, and professional values as competencies required for nursing graduates [15]. To understand the perception of competence among new nursing graduates, Ramritu and Barnard [8] conducted a qualitative study with six recently graduated nurses who had practiced for three months. Eight themes emerged, describing competence as the following: safe practice, limited independence, utilization of resources, management of time and workload, ethical practice, performance of clinical skills, knowledge, and evolving. Based on our review of the literature, we determined the definition of nurse competence covers the domains of knowledge, clinical skills, clinical reasoning and judgment, and professional behavior.

In the next step of developing the Clinical Competence Questionnaire, we created a list of nursing competencies with consideration to the definitions of nursing competence reported in the literature. Our list was based on literature reviews [5, 9, 10, 16] and on our own knowledge of the clinical skills that new nursing graduates need for entry into practice. From the literature reviews, we identified 50 items of nurse competence evaluation for upcoming baccalaureate nursing graduates. We then conducted focus groups with 16 clinical instructors who provided their opinions regarding the content of nursing competence. The focus groups generated 30 more items, making a total of 80 potential competence items for our instrument. We conducted discussions again with the clinical instructors to review the 80 potential items and determine specific items regarded as necessary competencies that upcoming baccalaureate nursing graduates must possess in their clinical novice stage. From the initial 80 potential competency items, the group selected 47 items for further validation.

The preliminary Clinical Competence Questionnaire consists of 47 items that represent competencies categorized in either nursing professional behaviors (Items 1-16) or skills (Items 17-47, see Table 1). The instrument uses a 5-point Likert scale to measure the clinical competence level of upcoming baccalaureate nursing graduates. The scale's item response scores range from 1 (*do not have a clue*) to 5 (*know in theory, competent in practice without any supervision*). Total scores range from 47 to 235, with a higher score indicating a higher level of competence. The Clinical Competence Questionnaire items evaluate nursing competence: safe care, professional ethics, assessment, critical thinking, collaboration and communication, basic nursing routines, and technical skills - all of which are considered as required entry-level clinical competencies for nursing graduates.

2.2 Phase II: Psychometric property tests of the clinical competence questionnaire

2.2.1 Design and participants

This phase involved a cross-sectional study design. Participants were recruited from all students enrolled in the 2-year RN-to-BSN program in Taiwan who were scheduled to graduate in 2 months. A total of 354 students were invited; 340 of them completed and returned the questionnaire for a 96% response rate.

2.2.2 Procedure

The proposal was reviewed and approved by the local Institutional Review Board (IRB) in Taiwan. We presented a verbal explanation of the study to the students and distributed packets that included a cover letter with a description of the study's purpose and participants' rights, a demographic questionnaire, and the Clinical Competence Questionnaire. Students were assured that they had the right to not answer questions, or even the entire questionnaire, that they did not feel comfortable with. All of the invited students understood that completing and returning their questionnaires indicated that they agreed to participate in the study. Students were provided a self-addressed stamped envelope to send back the completed questionnaire. All data were collected and managed anonymously and confidentially.

Table 1. The 47 Items Representing Competencies Measured in the Clinical Competence Questionnaire

**Nursing professional behaviors:**
1. Following health and safety precautions
2. Taking appropriate measures to prevent or minimize risk of injury to self
3. Taking appropriate measures to prevent or minimize risk of injury to patients
4. Preventing patients from problem occurrence
5. Adhering to the regulation of patients' and families' confidentiality
6. Demonstrating cultural competence
7. Adhering to ethical and legal standards of practice
8. Maintaining appropriate appearance, attire, and conduct
9. Understanding patient rights
10. Recognizing and maximizing opportunity for learning
11. Applying appropriate measures and resources to solve problems
12. Applying or accepting constructive criticism
13. Applying critical thinking to patient cares
14. Communicating verbally with precise and appropriate terminology in a timely manner with patients and families
15. Communicating verbally with precise and appropriate terminology in a timely manner with healthcare professionals
16. Understanding and supporting group goals
**Skill competencies:**
17. Taking a history for new admissions
18. Performing and documenting patient health assessment
19. Answering questions for patients or families
20. Educating patients or families with disease-related care knowledge
21. Charting and documentation
22. Developing care plan for patients
23. Performing shift report
24. Performing hygiene and daily care routines
25. Providing rest and comfort measures
26. Assessing nutrition and fluid balance
27. Assessing elimination
28. Assisting activities and mobility, and changing position
29. Providing emotional and psychosocial support
30. Performing venipuncture
31. Starting intravenous injections
32. Changing intravenous fluid bottle or bag
33. Administering intravenous medications (or into intravenous bags)
34. Administering intramuscular medications
35. Performing subcutaneous (or intracutaneous) injection
36. Administering oral medications
37. Administering blood transfusion
38. Performing urinary catheter insertion and care
39. Performing sterile techniques
40. Performing postural drainage and percussion, and oxygen therapy
41. Performing preoperation/postoperation care
42. Performing enema
43. Performing upper airway suction
44. Performing tracheotomy care
45. Performing nasogastric tube feeding and care
46. Performing chest tube care with underwater seal management
47. Performing wound dressing care

2.2.3 Data analysis

We managed and analyzed the study data using SPSS (Version 17.0; SPSS Inc., Chicago, IL). Norbeck [28] proposed that for a report on instrument development to be publishable, the report must address at least one type of content validity,

test-retest reliability, and internal consistency reliability, as well as at least one type of criterion-related validity and construct validity. Therefore, we tested the reliability of the Clinical Competence Questionnaire with Cronbach's alpha, inter-item correlation, and item-total correlation. We assessed the scale's validity by using content validity with expert panel reviews, concurrent validity for criterion-related validity, and factor analysis for construct validity.

To test the content validity of the Clinical Competence Questionnaire, we first conducted focus groups with clinical instructors to establish a list of possible competencies needed for upcoming baccalaureate nursing students. Subsequently, we asked a panel of three faculty experts [17] to review the specifications of the selected items and judge the degree of item significance to student competencies required for their first year of practice.

Testing for concurrent validity is useful for demonstrating an instrument's capability to predict subsequent events or behaviors [18]. To test the concurrent validity of the Clinical Competence Questionnaire, we simultaneously distributed the developed instrument not only to students in their final semester of a 2-year RN-to-BSN program, but also to a group of students in their second year of a 4-year traditional nursing program. We hypothesized that because students in the second year of a 4-year program have little experience in the medical-surgical practicum, they will have lower scores in clinical competence than students in the 2-year RN-to-BSN program.

Based on instrument-testing approaches recommended in the literature, we conducted a series of factor analyses to test the construct validity of the Clinical Competence Questionnaire. We applied the principal component factor analysis with oblique rotation to identify clusters of the Clinical Competence Questionnaire items that relate to the corresponding variables [17]. We also used parallel analysis, which compares eigenvalues extracted from raw data sets and eigenvalues from random data sets that parallel the raw data set, to determine the number of retained components [19, 20]. All components that had eigenvalues from raw data sets higher than eigenvalues from random data sets were retained [21]. We then performed principal component factor analysis by requesting the number of components to the number of components determined by the parallel analysis [19]. The criterion for considering a salient factor loading was .30 [22]; however, we considered cross-loading when an item loaded on more than one component at .32 or higher and deleted items cross-loaded strongly (equal or higher than .50) in two or more components [23]. The adequacy of the study's sample size was tested with the Kaiser-Meyer-Olkin measure [24].

3 Results

3.1 Descriptive results

The participants were students who were enrolled in the 2-year RN-to-BSN program in Taiwan and who were scheduled to graduate in 2 months. All of the students had completed their required clinical practicum hours. Of the 354 students who were invited to take part in the study, 340 agreed to participate (96% response rate). The mean age of participants was 22.8 years old (range = 21 to 32 years old). The participants had passed their clinical practicum, which included medical-surgical, obstetric and gynecologic, pediatric, psychiatric, and community health practicums. A majority of the participants (86.7%) had entered the 2-year RN-to-BSN program immediately after graduating from a 5-year college-level nursing program, and 12.7% of the participants currently worked in healthcare-related jobs. Participants had a mean of 5.38 ($n = 47$, $SD = 17.32$, range 3-120, median = 29, mode = 12) months of experience working as a nurse. Because past experience of being a nurse might influence how students perceived their clinical competence, we excluded those students who had work experience for the reliability and validity tests.

3.2 Validity of the clinical competence questionnaire

3.2.1 Content validity

Content validity refers to "the sampling adequacy of items for the construct that is being measured" [17]. The content validity index (CVI) approach includes the item content validity index (I-CVI) and the scale content validity index

(S-CVI). We computed the I-CVI for each item on the Clinical Competence Questionnaire as the number of experts giving a rating of 3 (*quite agree*) or 4 (*highly agree*) divided by the total number of experts. The S-CVI is the percentage of total items rated by the experts as either 3 or 4 [25]. A CVI score of .80 or higher is considered acceptable [17]. Based on the CVI approach recommended by Polit and Hungler [17], we invited three experts to judge the relevance and importance of the 47 items on the instrument for pregraduate nurse competence, using a 4-point CVI rating scale. All items of the scale received a rating of 3 or 4 from the three experts, making the I-CVI rating for each item 1.0 and the S-CVI calculation 1.0. Therefore, the content validity of the Clinical Competence Questionnaire was confirmed.

3.2.2 Factor analysis

Table 2 presents a detailed description of validity measures from the factor analysis of the Clinical Competence Questionnaire according to competency item and category. For the 16 items in the nursing professional behaviors category (Items 1-16), the Kaiser-Meyer-Olkin measure was .95, indicating an adequate sample number [24]. Parallel analysis resulted in one component, which could explain 59.1% of the variance of competence in nursing professional behaviors. The Cronbach's alpha for the professional behaviors category was .95 with a mean item-total correlation of .73 (range = .70 to .81) and a scale mean of 66.20 (*SD* = 9.79, range 16 to 80).

For the 31 items in the skill competencies category (Items 17-47), the Kaiser-Meyer-Olkin measure was .96, indicating an adequate sample number [24]. Parallel analysis resulted in three components of skill competencies. 68.94 % of the variance of competence in clinical skills could be explained by requesting three components. Component 1 (core nursing skills) contained 15 skills with a mean of 61.53 (*SD* = 10.13, range = 15 to 75): Items 24-25, 28-29, 32-36, 38-39, 42-45, 47. Component 1's Cronbach's alpha was .97 with a mean item-total correlation of .73 (range = .70 to .81). Component 2 (general performance) comprised 10 skills with a mean of 38.62 (*SD* = 5.92, range = 15 to 50): Items 17-23, 26-27. The Cronbach's alpha for Component 2 was .93 with a mean item-total correlation of .74 (range = .67 to .79). Component 3 (advanced nursing skills) contained 6 skills with a mean of 20.98 (*SD* = 4.38, range = 7 to 30): Items 30-31, 37, 40-41, 46. The Cronbach's alpha for Component 3 was .90 with a mean item-total correlation of .74 (range = .62 to .81).

The three components of the skill competencies category were highly inter-correlated (range = .69 to .79). However, the principal component analysis indicated that the following items were simultaneously loaded into Components 1 and 2: Item 24 (performing hygiene and daily care routines); Item 25 (providing rest and comfort measures); Item 27 (assessing elimination); Item 28 (assisting activities and mobility, and changing position); and Item 29 (providing emotional and psychosocial support). Likewise, Item 22 (developing care plan for patients) and Item 23 (performing shift report) was loaded into Components 2 and 3, whereas Item 42 (performing enema), Item 43 (performing upper airway suction), and Item 44 (performing tracheotomy care) cross-loaded in Components 1 and 3. Based on Osborne and Costello's (2005) [23] suggestion, we deleted Item 25, which loaded strongly (> .5) on two components.

After deleting Item 25, 68.87 % of the variance of competence in clinical skills could be explained. Component 1 (core nursing skills) contained 14 skills with a mean of 57.32 (*SD* = 9.50, range = 14 to 70). Component 1's Cronbach's alpha was .96 with a mean item-total correlation of .79 (range = .69 to .86). Cronbach's alpha and mean item-total correlation of Component 2 and Component 3 did not change from those of the 47-item scale. Other cross-loaded items including Item 23, 24, and 43 that also showed strong loading (equal or higher than .45) were not deleted from the scale because of their importance. Item 23 and 24 were routine skills that need to perform daily. Item 43, while not a skill that was performed often in every unit, was important in its relation to patient life.

After deleting Item 25, we took a closer look and discussed meanings of the cross-loaded items. We decided to keep Items 22, 23, 24, 27, 28, and 29 in Component 2 because they are more similar to the other general performance skills in Component 2 than to the core nursing skills in Component 1 and the advanced nursing skills in Component 3. Items 42, 43, and 44 were retained in Component 1. Despite this adjustment, each category of the Clinical Competence Questionnaire was still validated.

Table 2. Statistical Data Analysis Measures of the Clinical Competence Questionnaire, According to Competency Item and Category

Numbered Item	Nursing Professional Behaviors	Components of Skill Competencies		
		C1 with item 25 / C1 without item 25	C2 with item 25 / C2 without item 25	C3 with item 25 / C3 without item 25
16	.84			
7	.83			
11	.83			
9	.81			
15	.80			
3	.80			
10	.78			
2	.78			
12	.76			
8	.76			
14	.74			
6	.74			
5	.74			
13	.71			
1	.68			
4	.68			
45		.93/.94		
36		.93/.94		
34		.90/.91		
32		.81/.82		
33		.79/.79		
39		.77/.78		
47		.70/.71		
38		.69/.69		
44		.67/.66		.41/.41
28		.65/.67	.42/.41	
35		.64/.64		
43		.63/.63		.44/.45
42		.56/.56		.37/.37
25		.54/-	.52/-	
29		.54/.55	.37/.35	
19			.81/.82	
18			.76/.77	
21			.75/.76	
22			.69/.70	.34/-
17			.68/.67	
23			.64/.65	.51/.49
20			.63/.64	
24		.46/.48	.56/.55	
26			.55/.54	
27		.37/.39	.52/.52	
37				.91/.93
30				.78/.80
46				.78/.79
40				.70/.71
41				.67/.67
31				.54/.53
Variance explained	59.10%	55.46%/55.29%	7.62%/7.70%	5.86%/5.88%
Mean ± *SD*	66.20 ± 9.79	61.53 ± 10.13 /57.32 ± 9.50	38.62 ± 5.92	20.98 ± 4.38
Cronbach's alpha	.95	.97/.96	.93	.90
Correlation between components				
Nursing professional behaviors	1.00			
C1: Core nursing skills	.76*/.74*	1.00		
C2: General performance	.78*/.78*	.79*/.78*	1.00	
C3: Advanced nursing skills	.51*/.51*	.69*/.70*	.70*/.70*	1.00

Note. C1 = core nursing skills; C2 = general performance; C3 = advanced nursing skills.

* $p < .001$

ISSN 1925-4040 E-ISSN 1925-4059

After completing the factor analysis of the Clinical Competence Questionnaire and adjusting for simultaneous loading of items, four competency components emerged: (a) the nursing professional behaviors, represented in Items 1-16; (b) the general performance, represented in Items 17-24, 26-29; (c) the core nursing skills, represented in Items 32-36, 38, 39, 42-45, and 47; and (d) the advanced nursing skills, represented in Items 30, 31, 37, 40, 41, and 46. Table 3 presents an overview of the Clinical Competence Questionnaire's four components and their corresponding competency items.

Table 3. Components and Corresponding Items of the Clinical Competence Questionnaire, Identified by Principal Component Analysis

Component	CCQ Item Number and Competency
Nursing professional behaviors (16 items)	1. Following health and safety precautions<br>2. Taking appropriate measures to prevent or minimize risk of injury to self<br>3. Taking appropriate measures to prevent or minimize risk of injury to patients<br>4. Preventing patients from problem occurrence<br>5. Adhering to the regulation of patients' and families' confidentiality<br>6. Demonstrating cultural competence<br>7. Adhering to ethical and legal standards of practice<br>8. Maintaining appropriate appearance, attire, and conduct<br>9. Understanding patient rights<br>10. Recognizing and maximizing opportunity for learning<br>11. Applying appropriate measures and resources to solve problems<br>12. Applying or accepting constructive criticism<br>13. Applying critical thinking to patient cares<br>14. Communicating verbally with precise and appropriate terminology in a timely manner with patients and families<br>15. Communicating verbally with precise and appropriate terminology in a timely manner with healthcare professionals<br>16. Understanding and supporting group goals
Skill competence: General performance (12 items)	17. Taking a history for new admissions<br>18. Performing and documenting patient health assessment<br>19. Answering questions for patients or families<br>20. Educating patients or families with disease-related care knowledge<br>21. Charting and documentation<br>22. Developing care plan for patients<br>23. Performing shift report<br>24. Performing hygiene and daily care routines<br>26. Assessing nutrition and fluid balance<br>27. Assessing elimination<br>28. Assisting activities and mobility, and changing position<br>29. Providing emotional and psychosocial support
Skill competence: Core nursing skills (12 items)	32. Changing intravenous fluid bottle or bag<br>33. Administering intravenous medications (or into intravenous bags)<br>34. Administering intramuscular medications<br>35. Performing subcutaneous injection<br>36. Administering oral medications<br>38. Performing urinary catheter insertion and care<br>39. Performing sterile techniques<br>42. Performing enema<br>43. Performing upper airway suction<br>44. Performing tracheotomy care<br>45. Performing nasogastric tube feeding and care<br>47. Performing wound dressing care
Skill competence: Advanced nursing skills (6 items)	30. Performing venipuncture<br>31. Starting intravenous injections<br>37. Administering blood transfusion<br>40. Performing postural drainage and percussion, and oxygen therapy<br>41. Performing preoperation/postoperation care<br>46. Performing chest tube care with underwater seal management

3.2.3 Known groups validity

To test for concurrent validity, we compared the 46-item Clinical Competence Questionnaire response scores from participants who had clinical experience (experienced group) and those who did not have clinical experience (no-experience group). The scores from the experienced group on the entire scale, general performance, core nursing skills, and advanced nursing skills were significantly higher than the scores from the no-experience group (see Table 4). However, scores from the nursing professional behaviors category did not differ between those two groups. It is understandable that experienced students perceived higher skill competencies, which include general performance, core nursing skills, and advanced nursing skills than no-experience students because clinical nursing skills require practices to be competent. Thus, the concurrent validity of the Clinical Competence Questionnaire was confirmed, indicating the instrument can adequately predict subsequent events or behaviors [18].

Table 4. Comparisons of Competencies by Experienced and No-experience Students

	No experience (n = 293)	Experienced (n = 47)	Mann-Whitney U	*Z*	*p*
Entire scale	183.12 ± 26.35	195.30 ± 28.60	4912.00	3.16	.002
Professional behaviors	66.20 ± 9.79	67.65 ± 9.54	6210.50	1.08	.28
Core skills	57.32 ± 9.50	61.62 ± 10.28	4675.50	3.54	< .001
General performance	38.62 ± 5.92	40.72 ± 6.60	5657.50	1.97	.049
Advanced skills	20.98 ± 4.38	25.31 ± 4.43	3479.00	5.46	< .001

3.3 Reliability of the clinical competence questionnaire

The Cronbach's alpha for the entire 47-item Clinical Competence Questionnaire was .98. The instrument's mean item-total correlation was .70 (range = .50 to .81). For the nursing professional behaviors category (Items 1-16), the Cronbach's alpha was .95 and the mean item-total correlation was .73 (range = .70 to .81). The correlation matrix of scale items showed that the mean inter-item correlation of each item ranged from .34 to .84. For the skill competencies category of the instrument (Items 17-47), the Cronbach's alpha was .97, and the mean item-total correlation was .72 (range = .55 to .82). The correlation matrix of scale items showed that the mean inter-item correlation of each item in the skill competencies category ranged from .23 to .83.

After deleting Item 25, the Cronbach's alpha for the entire 46-item questionnaire was .98. The instrument's mean item-total correlation was .69 (range = .50 to .81). The Cronbach's alpha, mean item-total correlation, and inter-item correlation of the nursing professional behaviors category of the questionnaire did not change from those of the 47-item scale. For the skill competencies category of the instrument (Items 17-47), the Cronbach's alpha and mean inter-item correlation did not change from those of the 47-item scale while range of item-total correlation became .55 to .81.

4 Discussion and recommendation

Our purpose for developing the Clinical Competence Questionnaire to measure students' perceived clinical competence was guided by the philosophy that learning is a continuous process that must avoid shortcuts, especially context of today's ever-changing and complicated healthcare system. Cultivating and improving nurses' competence levels is a step-by-step process that requires lifelong learning experiences. Therefore, we recommend that healthcare educators, administrators, and staff acknowledge learning as a continuous process and patiently guide new nursing graduates to help them develop their skills steadily and gradually. We believe this approach will create a win-win situation with less frustration for educators, new graduates, and healthcare administrators and staff. For this reason, we conducted our study to develop and test a self-assessment instrument that identifies and measures appropriate clinical competence needed for the first year of practice for upcoming baccalaureate nursing graduates.

The results from our study indicate that the Clinical Competence Questionnaire demonstrates good reliability and validity for measuring the upcoming baccalaureate nursing graduates' perceived clinical competencies. The Clinical Competence

Questionnaire is also a useful tool and is easy to administer for the self-assessment of nursing clinical competence. Factor analysis revealed that the Clinical Competence Questionnaire contains four main competency components with corresponding and specific competencies required for nursing pregraduates: (a) nursing professional behaviors, which includes 16 competencies; (b) general performance, which includes 12 competencies; (c) core nursing skills, which includes 12 competencies; and (d) advanced nursing skills, which includes 6 competencies (see Table 3).

A noteworthy finding in our study is that Items 30 (performing venipuncture), 31 (starting intravenous injections), 37 (administering blood transfusion), 40 (performing postural drainage and percussion, and oxygen therapy), 41 (performing preoperation/postoperation care), and 46 (performing chest tube care with underwater seal management) in the Clinical Competence Questionnaire were loaded together in the advanced nursing skills category and were regarded by pregraduates in the study as being different from other core nursing skills. The reason for this distinction may be that nursing students in current clinical placements often are not allowed sufficient opportunities to practice these skills and may not even be permitted to do so by the clinical sites because of safety concerns for care. Therefore, these skills were regarded by students as advanced and difficult skills to perform. This finding is consistent with previous studies [26, 27]. Although some nursing schools provide high-tech equipment that provides opportunities for students to learn and practice sophisticated skills at school, only real-world experiences can advance students' competencies in particular skills, such as the six competency items identified in the Clinical Competence Questionnaire's advanced nursing skills factor. Our finding supports the belief that competence develops over time and improves with experience in real-life situations. Thus, we recommend that nurse educators and healthcare facilities provide student nurses with opportunities to practice advanced nursing skills, as identified in the Clinical Competence Questionnaire, during their clinical practicum.

We based our development of the Clinical Competence Questionnaire on Benner's [13] "From Novice to Expert" model, which specified and focused the context that we investigated and formed a basis for interpreting our study's results. The process of psychometric testing of the instrument met Norbeck's [28] proposed validity and reliability requirements for a report of instrument development to be publishable. Nevertheless, the findings from our study are limited. The psychometric property test of the Clinical Competence Questionnaire was conducted only on nursing students in a 2-year RN-to-BSN program. Thus, study results may not be generalized to students in all nursing school programs, such as a traditional nursing program. Further tests for baccalaureate nursing pregraduates in different programs are needed to establish advanced validation of the Clinical Competence Questionnaire. In addition, this instrument was designed as a self-assessment tool that measures perceived clinical competence. The scale therefore is not appropriate to evaluate the performance-based competence of students. However, this scale can still provide nurse educators and administrators a preliminary way to understand the confidence of students and new nurse staff in their clinical performance. The instrument's emphasis is on evaluating competence at the clinical novice stage for pregraduation nursing students. Based on our study's findings, we recommend that clinical administrators extend the orientation period for new nursing graduates entering practice and provide opportunities for appropriate staff development that connects each level of clinical learning experiences, according to Benner's [13] "From Novice to Expert" model.

Acknowledgement and funding

We thank for the National Science Council in Taiwan for supporting the funding to conduct the study.

References

[1] Sportsman S. Competency education and validation in the United States: What should nurses know? Nursing Forum. 2010; 45(3): 140-149. http://dx.doi.org/10.1111/j.1744-6198.2010.00183.x

[2] Beyea SC, von Reyn LK, Slattery MJ. A nurse residency program for competency development using human patient simulation. Journal of Nursing Staff Development. 2007; 23(2): 77-82. http://dx.doi.org/10.1097/01.NND.0000266613.16434.05

[3] Burns P, Poster E. Competency development in new registered nurse graduates: Closing the gap between education and practice. The Journal of Continuing Education in Nursing. 2008; 39(2): 67-73. http://dx.doi.org/10.3928/00220124-20080201-03

[4] Berkow S, Virkstis K, Stewart J, Conway L. Assessing new graduate nurse performance. The Journal of Nursing Administration. 2008; 38(11): 468-474. http://dx.doi.org/10.1097/01.NNA.0000339477.50219.06

[5] Meretoja R, Isoaho H, Leino-Kilpi H. Nurse Competence Scale: Development and psychometric testing. Journal of Advanced Nursing. 2004; 47(2): 124-133. http://dx.doi.org/10.1111/j.1365-2648.2004.03071.x

[6] Watson R, Stimpson A, Topping A, Porock D. Clinical competence assessment in nursing: A systematic review of the literature. Journal of Advanced Nursing. 2002; 39(5): 421-431. http://dx.doi.org/10.1046/j.1365-2648.2002.02307.x

[7] King's College London. Nursing competence: What are we assessing and how should it be measured? Policy+: Policy Plus Evidence, Issues and Opinions in Healthcare. 2009: 18, 1-2. Available from http://www.kcl.ac.uk/content/1/c6/05/68/69/PolicyIssue18.pdf.

[8] Ramritu PL, Barnard A. New nurse graduates' understanding of competence. International Nursing Review. 2001; 48(1): 47-57. http://dx.doi.org/10.1046/j.1466-7657.2001.00048.x

[9] Raines DA, Lynn CE. Nursing practice competency of accelerated bachelor of science in nursing program students. Journal of Professional Nursing. 2010; 26(3): 162-167. http://dx.doi.org/10.1016/j.profnurs.2009.12.004

[10] Boxer E, Kluge B. Essential clinical skills for beginning registered nurses. Nurse Education Today. 2000; 20(4): 327-335. http://dx.doi.org/10.1054/nedt.1999.0415

[11] Utley-Smith Q. 5 competencies needed by new baccalaureate graduates. Nurse Education Perspective. 2004; 25(4): 166-170. PMid:15387509

[12] Meretoja R, Leino-Kilpi H. Instruments for evaluating nurse competence. Journal of Nursing Administration. 2001; 31(7/8): 346-352. http://dx.doi.org/10.1097/00005110-200107000-00005

[13] Benner P. From Novice to Expert: Excellence and Power in Clinical Nursing Practice. CA: Addison-Wesley. 1984.

[14] American Association of Colleges of Nursing. The Essentials of Baccalaureate Education for Professional Nursing Practice. 2008. Available from http://www.aacn.nche.edu/education/pdf/baccessentials08.pdf.

[15] Poster E, Adams P, Clay C, Garcia B, Hallman A, Jackson B, et al.. The Texas model of differentiated entry-level competencies of graduates of nursing programs. Nursing Education Perspectives. 2002; 26(1): 18-23.

[16] Fizgerald LM, Delitto A, Irrgang JJ. Validation of the clinical internship evaluation tool. Physical Therapy. 2007; 87(7): 844-860. http://dx.doi.org/10.2522/ptj.20060054

[17] Polit DF, Hungler BP. Nursing Research: Principles and Methods (6th ed.). Philadelphia: Lippincott. 1999. PMid:10482253

[18] Goodwin L. Changing conceptions of measurement validity: An update on the new standards. Journal of Nursing Education. 2002; 41(3): 100-106. PMid:11939227

[19] Patil VH, Singh SN, Mishra S, Donavan DT. Efficient theory development and factor retention criteria: Abandon the 'eigenvalue greater than one' criterion. Journal of Business Research. 2008; 61(2): 162-170. http://dx.doi.org/10.1016/j.jbusres.2007.05.008

[20] Lance CE, Butts MM, Michels LC. The sources of four commonly reported cutoff criteria: What did they really say? Organizational Research Methods. 2006; 9(2): 202-220. http://dx.doi.org/10.1177/1094428105284919

[21] O'Connor BP. SPSS and SAS programs for determining the number of components using parallel analysis and Velicer's MAP test. Behavior Research Methods, Instruments, & Computers. 2000; 32(3): 396-402. http://dx.doi.org/10.3758/BF03200807

[22] Munro BH. Statistical Methods for Health Care Research (4th ed.). Philadelphia: Lippincott. 2001.

[23] Osborne JW, Costello AB. Best practices in exploratory factor analysis: Four recommendations for getting the most from your analysis. Practical Assessment, Research & Evaluation. 2005; 10(7): 1-9.

[24] Kaiser HF, Rice J. Little Jiffy, Mark IV. Educational and Psychological Measurement. 1974; 34(1): 111-117. http://dx.doi.org/10.1177/001316447403400115

[25] Lynn MR. Determination and quantification of content validity. Nursing Research. 1986; 35(6): 382-385. http://dx.doi.org/10.1097/00006199-198611000-00017

[26] Marshburn DM, Engelke MK, Swanson MS. Relationships of new nurses' perceptions and measured performance-based clinical competence. The Journal of Continuing Education in Nursing. 2009; 40(9): 426-432. http://dx.doi.org/10.3928/00220124-20090824-02

[27] Casey K, Fink R, Krugman M, Propst J. The graduate nurse experience. Journal of Nursing Administration. 2004; 34(6): 303-311. http://dx.doi.org/10.1097/00005110-200406000-00010

[28] Norbeck J. What constitutes a publishable report of instrument development? Nursing Research. 1985; 34(6): 380-382. http://dx.doi.org/10.1097/00006199-198511000-00022
